# Life-threatening tracheal leiomyoma presenting as acute respiratory failure masquerading as refractory asthma: A case report

**DOI:** 10.1016/j.rmcr.2026.102494

**Published:** 2026-08-27

**Authors:** Nathaniel M. Ivanick, Marina Filice, Michelle Sabia, Jessica Baek, Khubaib Mohammad Murtafa

**Affiliations:** a Department of Thoracic Surgery, Roswell Park Comprehensive Cancer Center, Buffalo, NY, USA; b University of South Carolina, Columbia, SC, USA; c University at Buffalo, Department of Pulmonary and Critical Care, Buffalo, NY, USA

**Keywords:** Endobronchial leiomyoma, Airway obstruction, Diagnostic errors, Heuristics, Bronchoscopy

## Abstract

Primary tracheal tumors represent less than 1% of all pulmonary neoplasms, with endobronchial leiomyomas accounting for a minute fraction of benign airway lesions. Due to their slow growth and nonspecific clinical presentation, these tumors are frequently misdiagnosed as obstructive airway diseases, such as asthma. We report the case of a 27-year-old female with a 3-year history of progressive, treatment-resistant respiratory symptoms initially managed as severe asthma. Despite escalating medical therapy, her condition deteriorated, culminating in an emergency department presentation with fulminant acute respiratory failure. Chest computed tomography and subsequent rigid bronchoscopy revealed a smooth, well-encapsulated 1.9 cm pedunculated tracheal mass originating from the posterior membranous wall, causing 100% luminal obstruction. The tumor was successfully resected via bronchoscopic snare electrocautery and basket extraction, followed by argon plasma coagulation of the base. Histopathological and immunohistochemical evaluation confirmed a benign tracheal leiomyoma. Postoperatively, the patient experienced immediate, complete resolution of respiratory symptoms with no recurrence at her 3-month follow-up. Tracheal leiomyoma, though rare, can cause life-threatening central airway obstruction where timely imaging and bronchoscopic intervention are ultimately lifesaving.

## 1 Introduction

Primary tracheal tumors represent under 1% of all pulmonary neoplasms. Approximately two-thirds of these central airway tumors are malignant, while the remaining one-third are benign [1]. Within this benign cohort, endobronchial leiomyomas are exceptionally rare, accounting for less than 1% of tracheobronchial tumors [1]. Because these slow-growing lesions primarily manifest with progressive dyspnea and wheezing, they frequently mimic the protean symptoms of asthma [2]. Consequently, patients are often managed for refractory asthma, receiving ineffective long-term medical therapies while the underlying structural airway obstruction progresses unaddressed [2]. Medical heuristics is a rapid form of medical decision making that can be both positive (rapidly arriving at a likely diagnosis) and negative (refusal to reconsider the initially proposed diagnosis) are a well-known phenomenon [3]. In the case described below, diagnostic anchoring and a reliance on common clinical heuristics led to a critical delay in diagnosis, culminating in a near-catastrophic event for a young patient. While an initial working diagnosis of asthma in a 27-year-old non-smoking female is clinically reasonable, a sequential failure to respond to standard guideline-based therapy should trigger an immediate reconsideration of alternative etiologies. Despite multiple primary care visits, repeat emergency department evaluations, and an outpatient pulmonary consultation, the patient's diagnostic label remained unquestioned until she presented with fulminant acute respiratory failure. This case underscores the vital importance of utilizing a structured, stepwise approach to uncontrolled respiratory symptoms, highlighting that when common conditions prove refractory to treatment, clinicians must actively investigate structural mimics.

## 2 Case presentation

### 2.1 Outpatient history and clinical course

A 27-year-old never-smoking female, employed at a commercial flower nursery, was initially diagnosed with asthma at age 24 after presenting with a persistent cough and frequent wheezing. She was sequentially prescribed a short-acting beta-agonist (SABA) rescue inhaler and a budesonide inhaler. Over the subsequent three years, her respiratory symptoms progressed with frequent, severe exacerbations leading to multiple workplace absences. She was evaluated at multiple primary care visits and emergency department (ED) presentations, where she received systemic glucocorticoids and nebulized bronchodilators that yielded only transient symptomatic relief.

As her dyspnea worsened, the patient developed a persistent globus sensation and explicitly requested an otolaryngology evaluation, which was denied. She was subsequently referred to an outpatient pulmonologist; however, pulmonary function testing (PFT) was not performed, and no diagnostic modifications were made. In the weeks prior to her acute presentation, her dyspnea escalated to the point where she was unable to sleep for more than 30 consecutive minutes. She developed profound daytime fatigue, requiring an increase in SABA use to three to four times daily without therapeutic benefit. Her roommates reported the development of prominent jerking, tremor-like movements during her brief periods of sleep.

### 2.2 Acute presentation and emergency stabilization

The patient ultimately presented to an outside hospital ED in severe respiratory distress (*in extremis*), demonstrating severe diffuse wheezing and sentence-word dyspnea. On room air, she was profoundly hypoxic and was placed on supplemental oxygen via nasal cannula. Initial arterial blood gas and serum laboratory evaluation revealed severe respiratory acidosis and acute end-organ hypoperfusion (Table 1).

**Table 1:** Arterial Blood Gas (ABG) of the patient when she presented to Emergency Department (ED); pCO2- partial pressure of carbon dioxide; WBC- White blood cells; AST- Aspartate aminotransferase; ALT- Alanine aminotransferase.

Parameter	Patient Value
Arterial pH	7.18
pCO2	110 mmHg
WBC	19000/μL
AST	13322 U/L
ALT	1810 U/L

She was initially placed on bilevel positive airway pressure (BiPAP) with settings of 20/8 ${\text{cm}\;H}_{2}O$, which resulted in only marginal improvement of her hypercapnic respiratory acidosis. A computed tomography (CT) scan of the chest, abdomen, and pelvis was performed; the preliminary radiological report indicated clear lung fields but suggested possible gallbladder inflammation.

The patient was transferred to the intensive care unit (ICU) due to persistent, refractory respiratory acidosis. Her initial ICU vital signs revealed a heart rate of 135 bpm, a respiratory rate of 28 breaths/min, a blood pressure of 134/84 mmHg, and an ${\text{SpO}}_{2}$ of 97% on BiPAP. Physical examination demonstrated diffusely diminished breath sounds to auscultation. Empiric management was initiated with intravenous dexamethasone, nebulized bronchodilators, azithromycin, and piperacillin/tazobactam over joint concerns regarding her leukocytosis and potential acute cholecystitis.

**Fig. 1:**
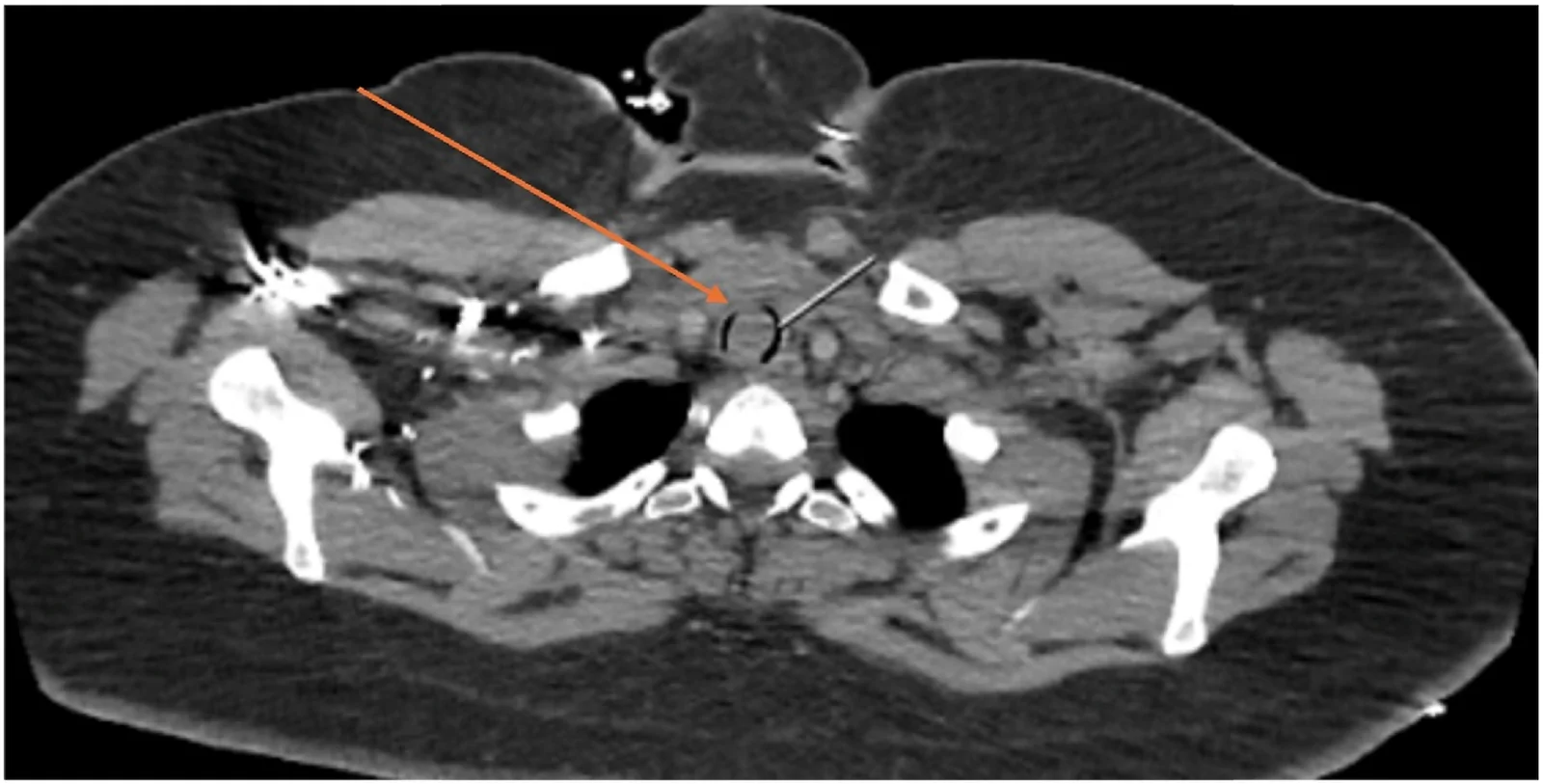
Axial chest computed tomography (CT) scan at the level of the thoracic inlet demonstrating a well-circumscribed, soft-tissue mass arising from the posterior membranous wall (arrow), resulting in critical, near-total mechanical obstruction of the tracheal lumen.

Upon formal comprehensive re-review of the chest CT by the radiology team, a well-defined soft tissue mass was identified arising from the posterior margin of the trachea at the level of the thoracic inlet, causing near-total luminal obstruction (Fig. 1).

### 2.3 Interventional management

Thoracic surgery and anesthesia teams were emergently consulted, and the patient was transferred to the operating room for anticipated difficult airway management, with preparations made for an emergent cricothyroidotomy or tracheostomy if necessary. Dynamic fiberoptic bronchoscopy was performed, visualizing a round, smooth, mucosal-covered mass in the upper trachea. She was successfully and safely intubated fiberoptically utilizing a 5.5 mm endotracheal tube (ETT) loaded over the bronchoscope.

**Fig. 2:**
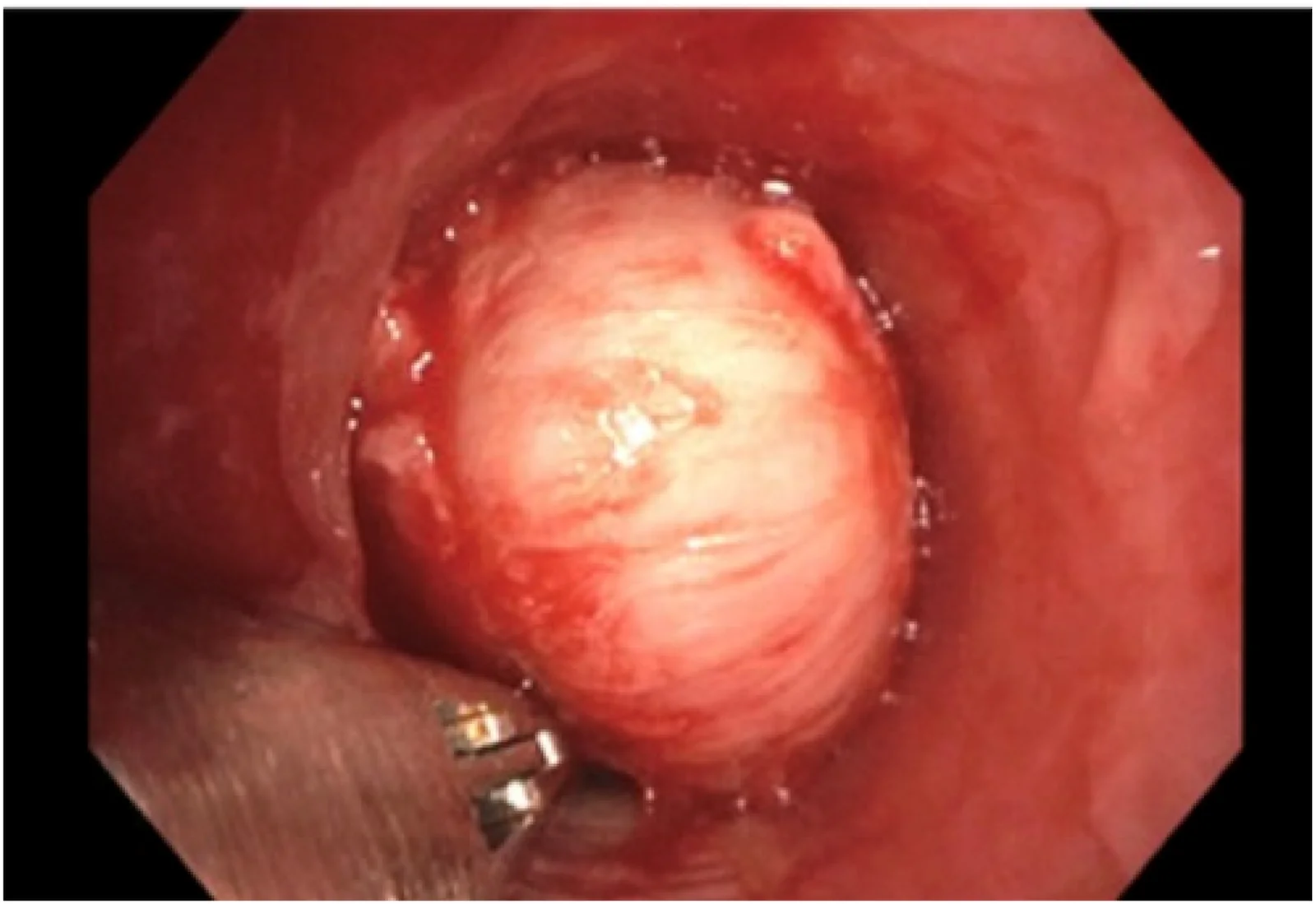
Direct visualization via rigid bronchoscopy confirming a massive, smooth, endotracheal tumor on a narrow, pedunculated stalk originating from the posterior membrane, causing near total mechanical distal airway occlusion.

**Fig. 3:**
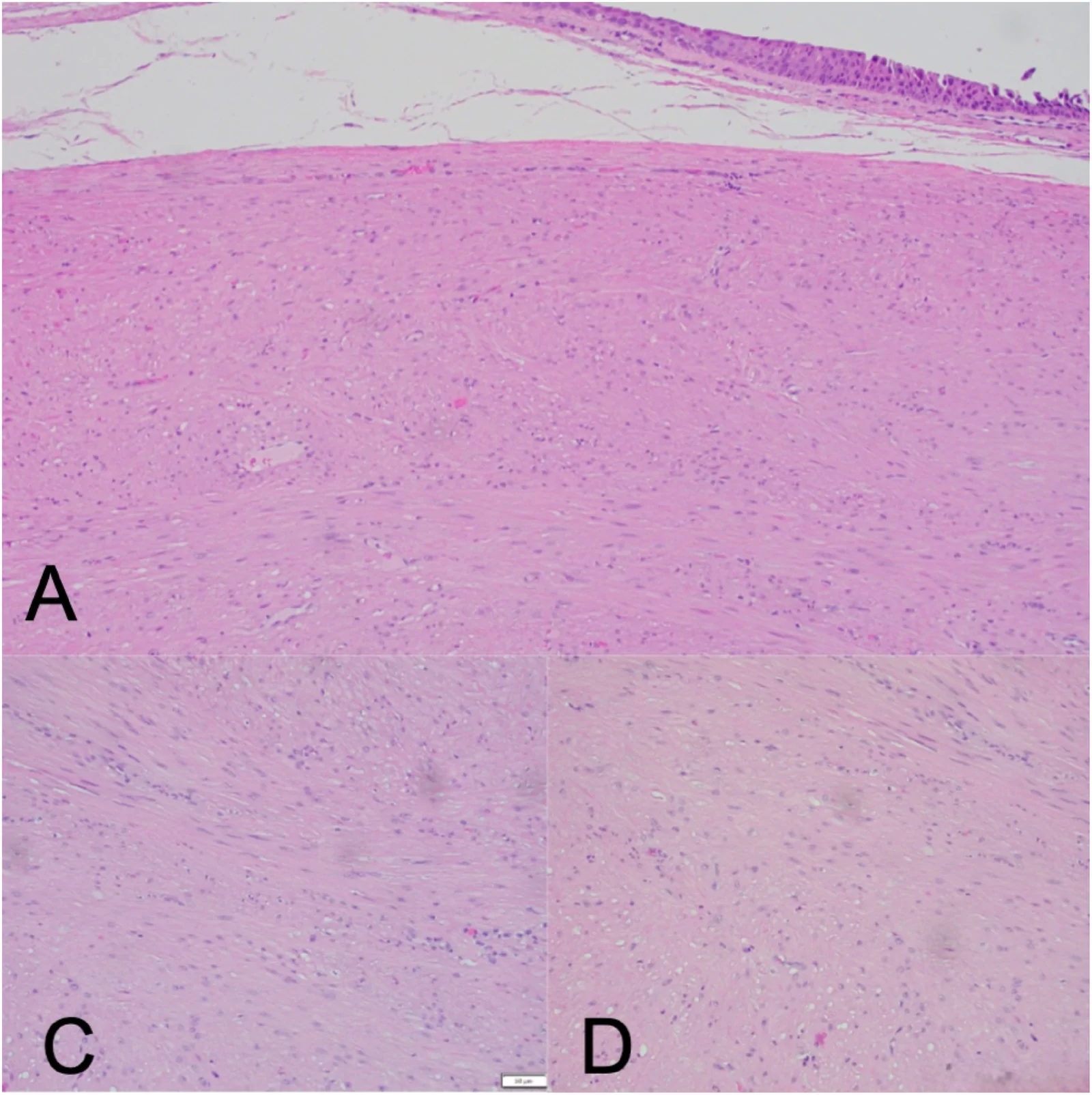
**Histopathological examination of the tracheal leiomyoma.** (A) Low-power hematoxylin and eosin (H&E, ×4) demonstrating a well-circumscribed submucosal spindle cell neoplasm beneath the respiratory mucosa. (B) Intermediate-power H&E (×10) showing intersecting fascicles of spindle cells characteristic of smooth muscle differentiation. (C) High-power H&E (×20) demonstrating bland spindle cell morphology with elongated nuclei, without cytologic atypia, mitotic activity, or tumor necrosis.

Following stabilization, the patient was transferred to our tertiary care center for definitive therapeutic airway intervention. Upon arrival in our ICU, she was mechanically ventilated but fully alert and able to communicate comfortably via a dry-erase board. The following day, she underwent rigid bronchoscopy, which revealed a massive endotracheal tumor on a narrow, pedunculated stalk originating from the membranous posterior tracheal wall, causing 100% mechanical obstruction of the distal airway (Fig. 2) (see Fig. 3).

Interventional pulmonology team successfully advanced a hot snare around the pedunculated stalk and performed snare electrocautery, freeing the lesion entirely from its attachment point on the posterior membrane. Given that the resected mass measured 19 mm in diameter-exceeding the average adult female tracheal diameter of approximately 18 mm-extraction through the vocal cords proved technically challenging. The mass was securely enclosed within a bronchoscopic basket and carefully removed across the vocal cords using controlled physical traction. Following complete specimen extraction, the residual base of the tumor stalk was safely ablated utilizing argon plasma coagulation (APC).

### 2.4 Histopathology and postoperative course

**Fig. 4:**
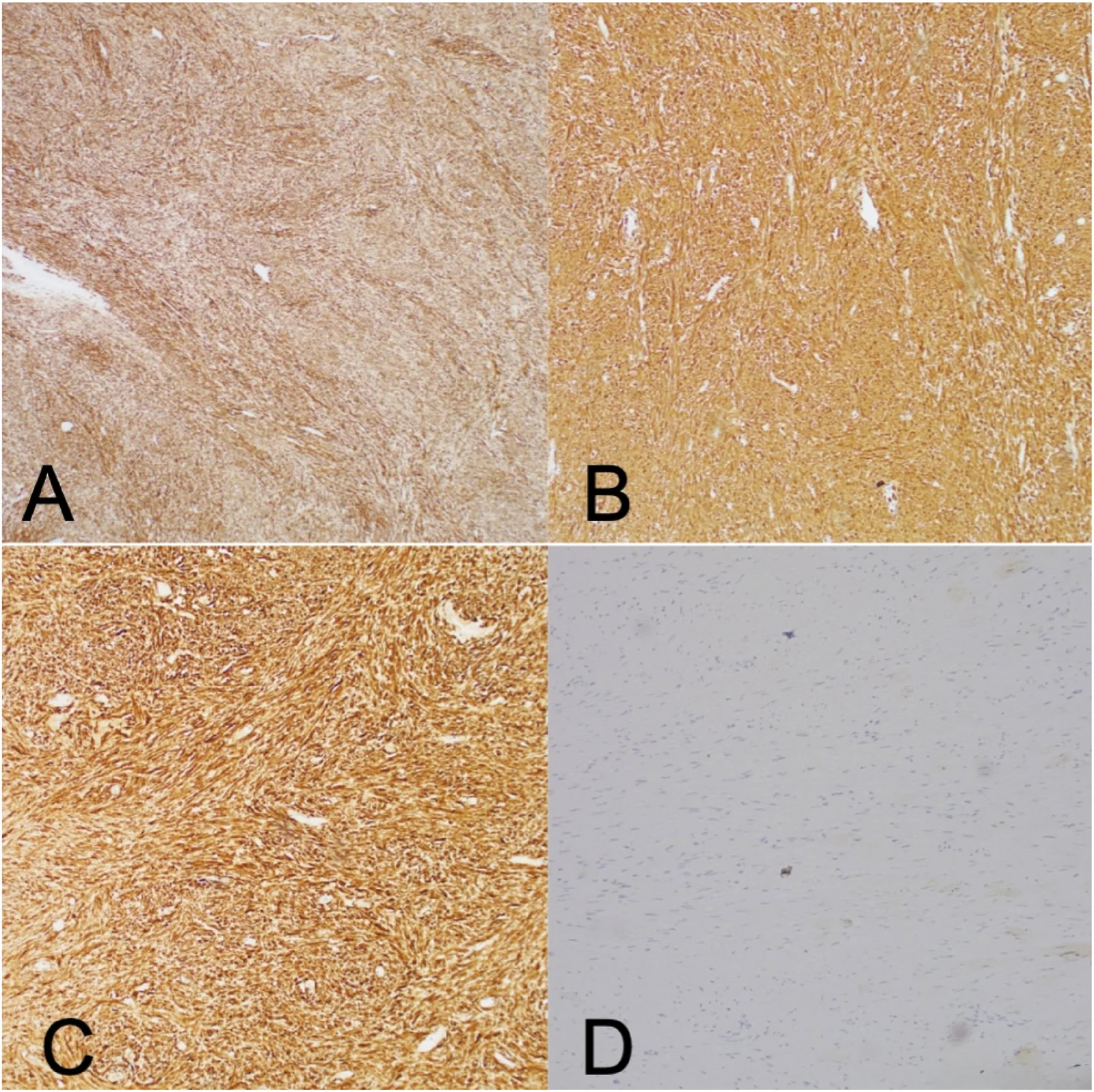
**Immunohistochemical characterization of the tracheal leiomyoma.** (A) Diffuse cytoplasmic positivity for desmin (×4). (B) Negative staining for S100 protein (×4). (C) Diffuse cytoplasmic positivity for h-caldesmon (×4), confirming smooth muscle differentiation. (D) Ki-67 immunostaining (×4) demonstrating an extremely low proliferative index (approximately 1%), supporting the diagnosis of a benign leiomyoma.

Gross pathology confirmed a 1.9 cm, well-encapsulated benign mesenchymal tumor. On immunohistochemical (IHC) profiling, the tumor cells demonstrated strong, diffuse positivity for Desmin, Caldesmon, and Smooth Muscle Actin (SMA). The Ki-67 proliferation index was exceptionally low at 1%, establishing a definitive diagnosis of a benign primary tracheal leiomyoma (Fig. 4).

The patient was successfully extubated the morning following the rigid bronchoscopy and was safely discharged home two days post-procedure. At her 1-month and 3-month follow-up outpatient appointments, she reported complete, permanent resolution of her dyspnea and cough, allowing for the complete cessation of all previous asthma therapies. A repeat surveillance bronchoscopy performed at 3 months post-procedure confirmed a completely healed tracheal mucosa with zero evidence of tumor recurrence. Longitudinal interval surveillance bronchoscopies are planned to monitor the site.

## 3 Discussion

Tracheal leiomyomas are benign mesenchymal neoplasms arising from the smooth muscle cells of the membranous posterior tracheobronchial tree, accounting for approximately 1% of all benign tracheal tumors [4]. This disease demonstrates a distinct 2:1 female-to-male predominance [4]. Notably, in female patients, primary pulmonary smooth muscle tumors may occasionally represent a manifestation of benign metastasizing leiomyoma (BML)- a rare syndromic condition where histologically benign uterine leiomyoma cells disseminate hematogenously to extrauterine organs, most frequently the lungs [5]. Histologically, leiomyomas are characterized by intersecting bundles of well-differentiated smooth muscle cells that characteristically express smooth muscle differentiation markers, including Desmin, Caldesmon, and smooth muscle actin (SMA) [6]. Distinguishing a benign endobronchial leiomyoma from a malignant leiomyosarcoma relies heavily on the mitotic rate and nuclear proliferation profiling. The utilization of the Ki-67 proliferation index is diagnostic in this differentiation; a significantly elevated Ki-67 index indicates high cellular turnover characteristic of sarcoma, whereas the exceptionally low Ki-67 index of 1% observed in this case definitively established the benign nature of the lesion [6].

The optimal therapeutic approach for endobronchial leiomyomas is primarily dictated by tumor size, structural morphology, and the extent of the attachment base along the posterior membrane. In our patient, the pedunculated morphology allowed for a safe and definitive endobronchial resection using rigid bronchoscopic snare electrocautery. Subsequent application of argon plasma coagulation (APC) provided safe thermal ablation of the residual mucosal margins to mitigate the risk of local recurrence. For tumors with broader, sessile bases, alternative bronchoscopic modalities such as cryoprobe extraction or laser tumor ablation are frequently required [7]. Surgical tracheal resection and reconstruction remain indicated if bronchoscopic intervention is unfeasible due to unfavorable tumor positioning, extensive transmural airway involvement, or localization within the peripheral lung parenchyma [4]. Current interventional algorithms recommend primary endobronchial resection as the initial treatment of choice, reserving surgical resection for cases of local recurrence or technically impossible endoscopic access [4]. The long-term prognosis for tracheal leiomyoma is excellent, with recurrence rates primarily dictated by the completeness of the initial margin resection. In a foundational case series of 16 patients, the authors reported three recurrences, two of which resulted directly from incomplete initial margins and one related to a technically complex position at the main carina [4].

Tracheal leiomyoma is an extraordinarily rare etiology of wheezing in a young, never-smoking adult and should understandably not be the primary working diagnosis during initial clinical encounters. While the medical adage "when you hear hoofbeats, think of horses, not zebras" serves as an essential framework for common diseases, clinicians must remain vigilant to the reality that rare conditions do exist. In this case, a young woman spontaneously developed severe, progressive asthma-like symptoms in her third decade of life—an atypical timeline for classic atopic asthma. Furthermore, her persistent complaints of a globus sensation represented a notable clinical red flag atypical of standard bronchospastic disease that should have prompted early structural investigation. Additional alternative diagnoses that warranted exploration given her clinical background included specialized inhalational or fungal exposures at the flower nursery, severe gastroesophageal reflux disease, or hypersensitivity pneumonitis.

The clinical teams involved in this patient's early care fell victim to classic cognitive pitfalls, specifically availability heuristics and anchoring bias [3]. Because asthma is highly prevalent among young adults, the diagnosis was readily assumed (despite the complete absence of confirmatory pulmonary function testing) and subsequently anchored upon. Clinicians continued to escalate guideline-based corticosteroid and bronchodilator therapies without success, treating the diagnostic label rather than the non-responsive patient. Furthermore, we hypothesize that the patient's demographic status as a young adult female may have implicitly compounded these cognitive heuristics, leading to a minimization of her atypical symptoms (such as the globus sensation). While making a definitive correlation with literature endpoints is complex, extensive healthcare research has investigated whether patient and provider sex differences influence the reliance on cognitive heuristics during medical decision-making [8,9]. On average, female primary care providers have been shown to dedicate more time to patient encounters, though clear, systematic trends regarding how provider gender impacts structural heuristic errors remain undefined [8,9]. This case serves as a powerful reminder that when a common respiratory diagnosis proves entirely refractory to appropriate guideline-directed therapy, clinicians must break the anchoring bias, step down the diagnostic heuristic, and actively investigate structural airway mimics.

## 4 Conclusion

Primary tracheal neoplasms and specifically endobronchial leiomyomas remain exceptionally rare clinical entities that are frequently misdiagnosed as asthma due to overlapping, non-specific respiratory symptoms. In this case, rigid adherence to an initial diagnostic label in the absence of objective confirmation or clinical response nearly resulted in a catastrophic outcome. This case serves as a critical reminder that when patients present with persistent, progressive, or treatment-refractory respiratory complaints, clinicians must actively look past cognitive anchoring biases. A structured, stepwise diagnostic re-evaluation—incorporating early physiological lung function testing, cross-sectional airway imaging, and frontline diagnostic bronchoscopy, is vital to ruling out life-threatening structural airway mimics and ensuring timely, definitive interventional care.

## CRediT authorship contribution statement

**Nathaniel M. Ivanick:** Writing – original draft, Supervision, Project administration, Methodology, Conceptualization. **Marina Filice:** Writing – review & editing, Visualization, Data curation. **Michelle Sabia:** Writing – review & editing, Methodology, Data curation. **Jessica Baek:** Writing – review & editing, Writing – original draft, Methodology. **Khubaib Mohammad Murtafa:** Writing – review & editing, Writing – original draft, Visualization.

## Ethics statement

Institutional Review Board approval was not required for this case report.

## Declaration of generative AI and AI-assisted technologies in the manuscript preparation process

During the preparation of this manuscript, the authors used ChatGPT to assist with language editing, grammar correction, and improvement of clarity and readability. After using this tool, the authors carefully reviewed and edited the content and take full responsibility for the accuracy, integrity, and originality of the work.

## Funding sources

This study was not supported by any sponsor or funder.

## Declaration of competing interest

The authors declare that they have no known competing financial interests or personal relationships that could have appeared to influence the work reported in this paper.
